# Molecular chaperone therapy- the future in cancer

**DOI:** 10.1186/1750-9378-7-20

**Published:** 2012-08-13

**Authors:** Abdul Moid Shehzad, Om Dawani, Shehryar Munir, Syed Anas Hussain

**Affiliations:** 1 Medical Student, Dow Medical College, Dow University of Health Sciences, Karachi, Pakistan

## 

Molecular chaperone or heat shock proteins (HSP) are vital proteins that increase cell survival by allowing it to combat stress caused by injurious stimuli through certain cyto-protective mechanisms [1]. These cyto-protective mechanisms of molecular chaperones, especially HSP 90 [2], have a negative effect designated to favor tumor growth and metastasis among breast cancer, leukemia, pancreatic and ovarian cancer [3,4].

Stabilization of the structure of important agents in malignant transformation, such as kinases (Src and Met tyrosine kinases) and transcription factors (e.g., hypoxia inducible factor, HIF1) allows molecular chaperones to stimulate angiogenesis by promoting endothelial cell proliferation and permitting growth of cancer beyond the oxygen capacity of tissue diffusion [5]. Molecular chaperones disrupt the programmed cell death pathway (apoptosis) by inducing mutant forms of tumor growth suppressors and DNA repair proteins (p53 and MSH2) [6-8]. New multi-target antineoplastic drugs like Geldanamycin, purine scaffold inhibitors, and Radicicol [9] have been developed to oppose all such activity of molecular chaperones.

The new therapeutic agents or Heat Shock Protein inhibitors function by blocking the intrinsic ATPase activity of molecular chaperones allowing oncogenic proteins (Raf-1, Akt/PKB, ErbB2, Cdk4, Polo-1, Met)[10] to be targeted by the ubiquitin proteasome pathway due to no chaperone protection [2,9]. An example is the positive result of the phase II clinical trial of HER2 positive breast cancer being treated by Hsp90 inhibitor 17-AAG followed with Trastuzumab [11]. Although directed towards distinct molecular targets, HSF inhibitors also inhibit other multiple cancer promoting signaling pathways, increasing the efficacy in treatment [12]. Synergistically usage of these new molecular chaperone inhibitors with standard chemotherapeutic drugs had positive results of tumor cell apoptosis and significant regression in treatment of leukemia and breast cancer respectively [13],

Despite effective results in phase 1 of clinical trials [14], HSP inhibitors cause reduction in stress-adaptive responses of normal cells leading to apoptosis [1]. Depletion of C2C12 (for muscle cell survival) by Geldanamycin derivatives [15] and colon adenocarcinoma growth during 17AAG treatment are some of the examples of this adverse effect [16]. However, greater affinity of HSP inhibitors towards tumoral chaperones specifically, is a reason that many clinical trials have not reported this side effect, for example 17AAG has 100 times greater affinity for tumoral versus normal cell HSP90 [1,17].

Although still in phase 2 of clinical trial, the development of HSP inhibitors provides an exciting alternative for molecular-based therapy in cancer [18]. HSP inhibitors like Gantespib, have shown a more promising future with a broader spectrum against various malignancies and better safety advantages in comparison to first and second generations HSP inhibitors [19]. Overall the advanced mechanism-based use of HSP inhibitors, both alone and in combination with other drugs, should help in the improvement of treatment of multiple forms of cancer in the future with minimal side effects.
